# Optimum Combination of Insulin-Transferrin-Selenium and Fetal Bovine Serum for Culture of Rabbit Articular Chondrocytes in Three-Dimensional Alginate Scaffolds

**DOI:** 10.1155/2009/747016

**Published:** 2009-05-27

**Authors:** Lanlan Zhang, Hong Song, Xiaojun Zhao

**Affiliations:** Institute for Nanobiomedical Technology and Membrane Biology, West China Hospital, Sichuan University, 610041 Sichuan, China

## Abstract

Fetal bovine serum (FBS) has been reported to affect chondrocyte biosynthesis in monolayer culture. Insulin-Transferrin-Selenium (ITS) was investigated as a partial replacement for FBS during in vitro culture of rabbit articular chondrocytes in three-dimensional alginate scaffold. Chondrocyte-seeded alginate hydrogels were cultured in Dulbecco's modified Eagle's medium plus 10% FBS, 1% ITS plus 2% FBS, 1% ITS plus 4% FBS, or 1% ITS plus 8% FBS. At designed time point, the Chondrocyte-seeded alginate hydrogels were harvested and evaluated with histological staining, immunohistochemistry, and quantitative gene expression analysis. Viable cell density and cell division were also evaluated. Chondrocytes biosynthesis and cell division in 1% ITS with 2% FBS medium were similar to that in medium added with 10% FBS. For a total culture of 3 weeks, phenotypic gene expression in chondrocyte-seeded hydrogels was maintained at high levels in medium with 1% ITS plus 2% FBS, while it was decreased to varying degrees in the other groups. In conclusion, with 1% ITS, medium with 2% FBS could promote chondrocyte biosynthesis and cell division, and prevented cell dedifferentiation in three-dimensional alginate scaffolds.

## 1. Introduction

Chondrocytes cultured in monolayer have a tendency to dedifferentiate [1, 2], and serial expanded chondrocytes in monolayer culture gradually dedifferentiated by reducing the production of type II collagen [3, 4]. Thus, to maintain the phenotypes of chondrocytes, three-dimensional scaffolds were developed for tissue engineering [5–7]. Alginate hydrogel is a frequently used scaffold for cartilage engineering. After long-term immobilization culture, rabbit articular chondrocytes in alginate gel still exhibited metabolic activities, patterns of division, as well as synthesis and secretion of extracellular matrix macromolecules such as type II collagen and proteoglycans [8]. Allogenic implants of chondrocytes in alginate gels was tested for the in vivo reconstruction of artificially full-thickness-damaged articular rabbit cartilage [9]. Thus, in this study, alginate was selected as a three-dimensional scaffold.

Currently, basal culture medium supplemented with 10% FBS was used for enhancing cell activities during in vitro three-dimensional culture of chondrocytes. However, it was reported that FBS would result in excessive cell proliferation [10] or chondrocyte phenotypic instability [11]. Studies have shown that Insulin-Transferrin-Selenium (ITS) prevented human chondrocyte dedifferentiation and promoted the formation of high-quality tissue engineering human hyaline cartilage [12]. ITS was used as a baseline medium supplement for in vitro studies concerning cartilage explants, as well as chondrocyte expansion or redifferentiation [13, 14]. However, the optimum combination of Insulin-Transferrin-Selenium and fetal bovine serum for culture of rabbit articular chondrocytes in three-dimensional alginate scaffolds has not been extensively investigated.

In this study, it was hypothesized that a small volume of FBS in medium with 1% ITS can sustain extracellular matrix synthesis and maintain the biological activities of chondrocytes in alginate hydrogels. In order to choose an appropriate FBS concentration, culture medium supplemented with 1% ITS plus 2% FBS, 1% ITS plus 4% FBS, 1% ITS plus 8% FBS, or medium plus 10% FBS were selected for three-dimensional culture of rabbit chondrocytes. The biological, biochemical properties of chondrocytes seeded in alginate hydrogels were evaluated with histological staining, immunohistochemistry, quantitative gene expression analysis.

## 2. Materials and Methods

### 2.1. Culture Medium

The basal medium was high-glucose Dulbecco's modified Eagle's medium (DMEM; Gibco), with ascorbate (50 *μ*g/mL), penicillin (100 U/mL), and streptomycin (100 *μ*g/mL). Basal medium was added with 1% Insulin-Transferrin-selenium (ITS; Gibco) plus 2% fetal bovine serum (FBS; Invitrogen), 1% ITS plus 4% FBS, 1% ITS plus 8% FBS, or 10% FBS for the in vitro culture of chondrocyte-seeded hydrogels.

### 2.2. Isolation and Culture of Chondrocytes

Chondrocytes were isolated from 4-week-old male New Zealand rabbits. The rabbits were sacrificed by an overdose anesthesia. The knee, hip, elbow joint cartilage slices were harvested, minced, and then washed three times in PBS. The fragments were digested in 0.25% trypsin for 30 minutes at 37°C, followed by digestion in 0.2% (w/v) collagenase II for 3 hours. After digestion, the suspension was filtered with 70 *μ*m stainless steel mesh. The cells released in the supernatant were collected by centrifuging (100 g/min, 5 minutes). Isolated chondrocytes were washed three times and counted in a hemocytometer by trypan blue dye exclusion. Chondrocytes were suspended in DMEM with 10% FBS and 100 U/mL penicillin and 100 *μ*g/mL streptomycin, 50 *μ*g/mL ascorbate and cultured in flasks at an initial density of 4 × 10^4^ cells/*c* *m* ^2^. The chondrocytes were incubated at 37°C, 5% *CO*_2_, and 95% humidity, and the medium was changed every three days. The cells were harvested and subcultivated at 80% confluence.

A 1.5% (w/v) alginate (Sigma) solution in 0.01 M PBS was prepared and sterilized by filtering through a 0.22 *μ*m pore size polyethersulfone filter (Millipore). The second-generation chondrocytes were used for seeding in the 1.5% alginate solution at a density of about 5 × 10^6^ cells/mL. For alginate scaffold encapsulation, isolated chondrocytes were resuspended in 1.5% alginate solution. The chondrocyte-alginate suspension was pipetted into 8 mm diameter silanized plastic rings (150 *μ*L/ring) in 24-well plates and gelled for 10 minutes by adding an excess volume of 102 mM CaCl_2_, and then the ring and the CaCl_2_ solution were removed. As designed, various media were added to the 24-well plates and placed in a cell culture incubator. Thereafter, the media were changed every three days.

### 2.3. Morphology of Chondrocytes in Alginate Hydrogels Cultured under Various Media

The morphology and proliferation of chondrocytes in alginate hydrogels cultured under various media were examined using an inverted phase contrast microscope (Olympus).

### 2.4. Proliferation Assays

The cellular activity of chondrocytes in the cultured scaffolds was estimated by MTT (3-(4,5-dimethylthiazol-2-yl)–2,5-diphenyltetrazolium bromide) assay. In this study, a modified MTT assay was employed according to Zund et al. report [15]. In brief, at different time points, 800 *μ*L serum-free medium and 80 *μ*L MTT solution (5 mg/mL in PBS) were added to each sample, followed by incubation at 37°C for 4 hours for MTT formazan formation; and then the medium was removed and the converted dye was dissolved with 800 *μ*L 10% sodium dodecyl sulfate (SDS) in 0.01 M HCl. After all crystals were solubilized, the optical density of the solution was determined at 570 nm against SDS solution as blank using an ELISA plate reader. Four parallel replicates were read for all samples.

### 2.5. Histology and Imunohistochemistry

Cell-seeded hydrogels were harvested at week 1 and week 3. The harvested samples were fixed in 4% paraformalydehyde in PBS overnight at 4°C, then washed, dehydrated in graded ethanol, cleared in xylene, and embedded in paraffin. Sections of 6 *μ*m were made through the cross-section of the sample and prepared for histological and immunohistochemical examination. Some of the sections were stained with Toluidine Blue dye solution.

For immunohistochemical analysis, immunohistochemistry was carried out to detect the expression of chondrocyte marker type II collagen. Sections were treated with 0.4% pepsin for 30 minutes at 37°C, washed with PBS, treated with 0.6% *H*_2_ *O*_2_ in methanol, and rinsed again with PBS. The immunohistochemical staining for collagen was then performed with rabbit type II collagen antibody (Calbiochem, 1 : 1000 in PBS) over night at 4°C. The following manipulations were carried out using Streptavidin/ Peroxidase (SP) staining kit (SP-9002, Zhongshan Golden Bridge Biotechnology Co., Ltd) according to the manufacturer's instructions and finally the color was developed with diaminobenzidine (DAB-kit, Vector). Cell nuclei were counterstained with Mayer's hematoxylin (Sigma) and embedded on microscopic slides using neutral balsam (Sigma) for microscopic examination. During the whole procedure of the experiment, the manipulations were strictly controlled and the time of staining was same for each sample.

### 2.6. Quantitative Gene Expression Analysis by Semiquantitative Real-Time PCR

Semiquantitative real-time PCR was performed to determine the gene expression for chondrocyte differentiation (type II collagen and aggrecan core protein), and dedifferentiation (type I collagen). At week 1 and week 3, total RNA in the chondrocyte-seeded hydrogels was extracted by Trizol reagent (Gibco) according to the manufacture's instruction. The RNA sample was converted to a complimentary DNA (cDNA) with RevertAid^TM^ First Strand cDNA Synthesis Kit (Fermentas) according to the manufacture's recommendation. Primers of rabbit Glyceraldehyde-3-phosphatase dehydrogenase (GAPDH, housekeeping gene), collagen type I, collagen type II, and Aggrecan core protein were designed with Primer Express v 3.0 software (Applied Biosystems) and blasted with GeneBank database sequences to get highly specific primers. The primers sequences were shown as follows:

Collagen type II: forward: 5′-CAGGATGTCCAGGAGGCT-3′, reverse: 5′-GCAGTGGCGAGGTCAGTAG-3′;
Collagen type I: forward: 5′-GCAACTTGAACAAGGCTGTC-3′,reverse: 3′-CAAGGAAGGGCAAACGAG-5′;
Aggrecan: forward: 5′-CGAGAATCAAATGGAGCCG-3′,reverse: 3′-CACAACACCTTTCACCACGAC-5′;
GAPHD: forward: 5′-ATGTTTGTGATGGGCGTGAA-3′,reverse: 5′-TGGAGGCAGGGATGATGTT-3′.

Semiquantitative real-time PCR was performed in a 25 *μ*L reaction system, and was programmed to run 35 cycles. The end products were separated with 1.5% agarose gel electrophoresis and the results were analyzed with UVI image software after capturing by a gel image system (Bio-Rad).

### 2.7. Statistical Evaluation 

All numerical data were presented as mean ± standard error of the mean (SEM). Difference between groups was evaluated by the student's *t*-test. *P* < .05 was statistically significant.

## 3. Results

### 3.1. Morphology of Chondrocytes in Alginate Hydrogels Cultured under Various Media

In order to reveal the morphology of cells cultured on three-dimensional alginate hydrogels under different media, cells cultured for 7, 14, and 21 days were examined.

No significant differences were observed between chondrocytes cultured under the four kind of tested media. It seems that the growth pattern, cell aggregations, and the rates of colonization between the tested groups were similar from the very beginning to the end of the culture periods. Chondrocytes in alginate hydrogel cultured under different media could be seen to form cell islets and cell aggregates with spherical shape, which maintained typical chondrocyte appearance (data not shown).

### 3.2. Proliferation Assays

**Figure 1:**
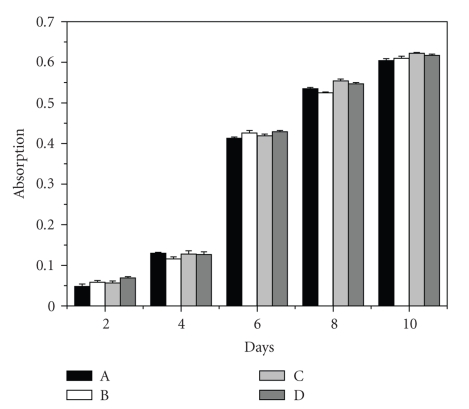
Chondrocyte proliferation in alginate hydrogels cultured in (a) medium supplemented with 1% ITS plus 2% FBS, (b) medium with 1% ITS plus 4% FBS, (c) medium with 1% ITS plus 8% FBS, or (d) medium with 10% FBS after various culture times. Values represent the mean and standard deviation, *n* = 6.

MTT assay was performed to measure the cell viability. Figure 1 shows the cell viability of chondrocytes cultured with different media at various time points, there was no significant difference (*P* > .05) between four groups. In addition, the cell viabilities of chondrocytes in all groups increased with culture time and the chondrocytes began to enter Log phase at about day 6.

### 3.3. Histological and Immunochemical Analysis

**Figure 2:**
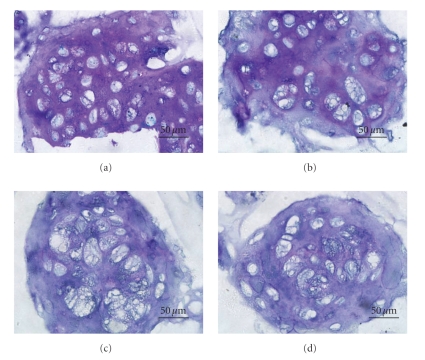
Toluidine blue staining for chondrocyte-seeded alginate hydrogels maintained in (a) medium supplemented with 1% ITS plus 2% FBS, (b) medium with 1% ITS plus 4% FBS, (c) medium with 1% ITS plus 8% FBS, or (d) medium with 10% FBS for 21 days. The scale bars indicate 50 *μ*m.

Production of glycosaminoglycans (GAGs) by chondrocytes in alginate hydrogels cultured under various media was confirmed by Toluidine Blue staining (Figure 2). For chondrocyte-seeded alginate hydrogels cultured in medium supplemented with 1% ITS plus 2% FBS for 21 days, significant staining for clusters of chondrocytes and their extracellular matrix was noted, indicating large amounts of GAG accumulation in the matrix surrounding chondrocytes. The groups cultured in the medium with 8% and 10% showed the lowest staining, which appeared to be significantly different from the others.

**Figure 3:**
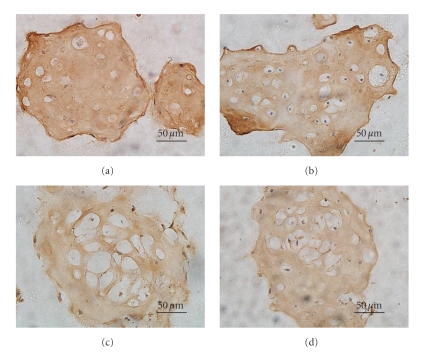
Immunohistochemical staining for chondrocyte-seeded alginate hydrogels maintained in (a) medium supplemented with 1% ITS plus 2% FBS, (b) medium with 1% ITS plus 4% FBS, (c) medium with 1% ITS plus 8% FBS, or (d) medium with 10% FBS for 21 days. The scale bars indicate 50 *μ*m.

Immunohistochemical staining of chondrocytes in alginate hydrogels cultured under various media is seen in Figure 3. Staining for collagen type II revealed significant pericellular matrix staining for chondrocyte-seeded alginate hydrogels cultured in medium supplemented with 1% ITS plus 2% FBS and in medium with 1% ITS plus 4% FBS. Whereas, for the groups (Figures 3(c)  and 3(d)) cultured in medium supplemented with 1% ITS plus 8% FBS and in medium with 10% FBS, the staining was less positive. The data show that the medium supplemented with 1% ITS plus lower FBS is more propitious to chondrocyte production and deposition of extracellular matrix molecules such as type II collagen and GAG-containing proteoglycans.

### 3.4. Quantitative Gene Expression of Chondrocytes in Alginate Hydrogels under Various Media

**Figure 4:**
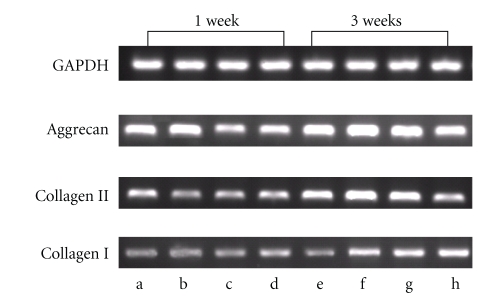
Agarose gel electrophoresis of the PCR products obtained from chondrocyte-seeded alginate hydrogel cultured in (a, e) medium supplemented with 1% ITS plus 2% FBS, (b, f) medium supplemented with 1% ITS plus 4% FBS, (c, g) medium supplemented with 1% ITS plus 8% FBS, or (d, h) medium with 10% FBS. Three target genes, aggrecan, type I, and II collagens were examined, while GAPDH was used as a housekeeping gene. Samples for lane a–d were cultured for 1 week and for lane e–h were cultured for 3 weeks.

Semiquantitative RT-PCR analysis showed collagen II, collagen I, and aggrecan expression of chondrocytes in alginate hydrogels cultured with different media (Figure 4) and the data were representative of five experiments. At week 1, the aggrecan expression of groups cultured in medium with 1% ITS plus 2% FBS, medium with 1% ITS plus 4% FBS was greater than the other tested groups; at week 3, aggrecan expression of the groups cultured in medium with 1% ITS plus 2% FBS, medium with 1% ITS plus 4% FBS, and medium with 1% ITS plus 8% FBS was slightly higher than that in medium with 10% FBS. At week 1, chondrocyte-seed alginate hydrogels cultured in medium with 1% ITS plus 2% FBS expressed the highest levels of type II collagen compared to the other three groups, and at week 4, the group cultured in medium with 10% FBS had the lowest expression level of type II collagen whereas those cultured in medium with 1% ITS plus 2% FBS, medium with 1% ITS plus 4% FBS, and medium with 1% ITS plus 8% FBS maintained a relatively high level of type II collagen expression. Besides, at week 3, chondrocyte-seeded hydrogels cultured in medium with 1% ITS plus 8% FBS and medium with 10% FBS expressed relatively high levels of type I collagen, indicating a loss of chondrocyte phenotype. By contrast, those cultured in medium with 1% ITS plus 2% FBS maintained low levels of type I collagen expression. These results suggested that medium with 1% ITS plus 2% FBS was sufficient to maintain a chondrocyte phenotype in alginate hydrogel.

## 4. Discussion

The final goal of this study was to find an optimum combination of ITS and fetal bovine serum for culture of rabbit articular chondrocytes in three-dimensional alginate scaffolds. Serum can provide nutrients for cell growth and promote cell synthesis and fetal bovine serum was frequently selected element for the culture of chondrocytes [15–18]. However, the addition of serum would cause chondrocytes to change its morphological features to become more fibroblastic and dedifferentiate in the monolayer culture [19, 20]. Moreover, medium supplemented with serum resulted in excessive tissue swelling and significant degradation of explant properties [21]. Recently, several reports [14, 22] have shown the potential of chondrocytes cultured either serum free or with a small volume of serum. The commercially available ITS was used in this study to support cell growth in low serum culture medium. It was demonstrated that medium supplemented with 1% ITS plus 2% FBS was able to enhance chondrocyte growth and reduced the dedifferentiation process of cultured chondrocytes. Since chondrocytes cultured with only 1% ITS medium had a relatively poor growth rate, we did not present these data.

As to serum-free or low-serum culture, most previous reports focused on monolayer. Since three-dimensional culture could maintain the phenotype for a long culture period, it is more practical to investigate the influence of serum chondrocytes cultured in three-dimensional scaffolds. It was proved that alginate scaffold biomaterial has a great potential in cartilage tissue scaffolds for cartilage tissue engineering [5, 8, 9, 23], thus we adopted alginate hydrogel as a three-dimensional scaffold.

In alginate hydrogel, chondrocytes distributed throughout the network of the matrices and maintained spherical morphology. For chondrocytes grown under the various culture media, significant differences were observed in the staining of Toluidine Blue and type II collagen immunohistochemistry. Compared with other tested groups, denser staining was shown for chondrocytes cultured in medium supplemented with 1% ITS plus 4% FBS as well as for the 1% ITS plus 2% FBS group. These results indicated that reducing the percent FBS may promote the maintenance of the chondrocyte phenotype to some extent. Results from semi-quantitative reverse transcriptase-polymerase chain reaction analysis also support this suggestion. At 7 days after cultivation, the mRNA for type II collagen and aggrecan was well expressed in all specimens and type I collagen was poorly expressed. However, after longer periods of culture, differences among the four groups were revealed; expression of type I collagen become evident for chondrocytes cultured under medium with 1% ITS plus 4% FBS, medium with 1% ITS plus 8% FBS, and medium with 10% FBS, while the expression level remained low for chondrocytes cultured in medium with 1% ITS plus 2% FBS.

In brief, 1% ITS supplementation combined with 2% FBS resulted in higher differentiation ability while high volume of FBS led to less extracellular matrix accumulation during long culture period. The reduction of the amount of animal serum used in the medium from 10% to 2% provided valuable implications on three-dimensional cartilage engineering. However, further work needs to be done to investigate the mechanical change on alginate hydrogel under this culture medium.

## 5. Conclusion

This study demonstrated that medium supplemented with 1% ITS plus 2% FBS could stimulate chondrocyte proliferation and increase cartilage matrix synthesis. Moreover, the presence of 1% ITS reduced the process of chondrocyte dedifferentiation. Chondrocytes seeded in alginate hydrogel could maintain their chondrocyte phenotype for long-period culture under medium supplemented with 1% ITS plus 2% FBS, which may be widely used in three-dimensional chondrocytes culture for tissue engineering.
